# 2-((*E*)-{[4-(Hy­droxy­meth­yl)phen­yl]imino}­meth­yl)phenol

**DOI:** 10.1107/S1600536812019368

**Published:** 2012-05-05

**Authors:** Shaaban K. Mohamed, Antar A. Abdelhamid, Mehmet Akkurt, Phillip E. Fanwick, A. M. Maharramov

**Affiliations:** a Chemistry and Environmental Division, Manchester Metropolitan University, Manchester M1 5GD, England; b Department of Physics, Faculty of Sciences, Erciyes University, 38039 Kayseri, Turkey; c Department of Chemistry, Purdue University, W. Lafayette, IN 47907, USA; d Department of Organic Chemistry, Baku State University, Baku, Azerbaijan

## Abstract

The title compound, C_14_H_13_NO_2_, adopts the enol–imine tautomeric form, with an intra­molecular O—H⋯N hydrogen bond which generates an *S*(6) ring motif. The dihedral angle between the aromatic rings is 7.85 (7)°. The crystal structure is stabilized by O—H⋯O, O—H⋯N and C—H⋯O hydrogen bonds, forming a two-dimensional array that stacks along the *a* axis. In addition, a C—H⋯π inter­action contributes to the stabilization of the crystal packing.

## Related literature

For background to Schiff base compounds, see: Elena *et al.* (2000 ▶); Mohamed *et al.* (2006 ▶); Rajavel *et al.* (2008 ▶); Uğraş *et al.* (2006 ▶); Wadher *et al.* (2009 ▶). For similar structures, see: Deveci *et al.* (2008 ▶); Karadayı *et al.* (2003 ▶); Koşar *et al.* (2010 ▶); Ünver *et al.* (2002 ▶). For the graph-set analysis of hydrogen bonding, see: Bernstein *et al.* (1995 ▶).
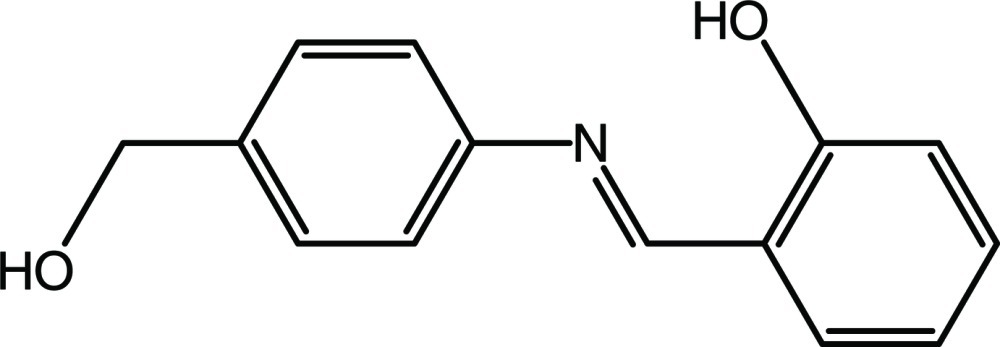


## Experimental

### 

#### Crystal data

C_14_H_13_NO_2_
*M* *_r_* = 227.25
Monoclinic, 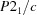

*a* = 19.8172 (14) Å
*b* = 4.7217 (1) Å
*c* = 12.3106 (2) Å
β = 104.005 (7)°
*V* = 1117.67 (9) Å^3^
*Z* = 4
Cu *K*α radiation
μ = 0.73 mm^−1^
*T* = 150 K
0.25 × 0.20 × 0.08 mm

#### Data collection

Rigaku RAPID II diffractometer
Absorption correction: multi-scan (*CrystalClear*; Rigaku/MSC, 2001 ▶) *T*_min_ = 0.838, *T*_max_ = 0.944
10711 measured reflections
1958 independent reflections
1641 reflections with *I* > 2σ(*I*)
*R*_int_ = 0.030

#### Refinement

*R*[*F* ^2^ > 2σ(*F* ^2^)] = 0.037
*wR*(*F* ^2^) = 0.107
*S* = 1.14
1958 reflections
163 parameters
H atoms treated by a mixture of independent and constrained refinement
Δρ_max_ = 0.23 e Å^−3^
Δρ_min_ = −0.18 e Å^−3^

### 

Data collection: *CrystalClear* (Rigaku/MSC, 2001 ▶); cell refinement: *CrystalClear*; data reduction: *CrystalClear*; program(s) used to solve structure: *SHELXS97* (Sheldrick, 2008 ▶); program(s) used to refine structure: *SHELXL97* (Sheldrick, 2008 ▶); molecular graphics: *ORTEP-3 for Windows* (Farrugia, 1997 ▶) and *PLATON* (Spek, 2009 ▶); software used to prepare material for publication: *WinGX* (Farrugia, 1999 ▶) and *PLATON*.

## Supplementary Material

Crystal structure: contains datablock(s) global, I. DOI: 10.1107/S1600536812019368/tk5090sup1.cif

Structure factors: contains datablock(s) I. DOI: 10.1107/S1600536812019368/tk5090Isup2.hkl

Supplementary material file. DOI: 10.1107/S1600536812019368/tk5090Isup3.cml

Additional supplementary materials:  crystallographic information; 3D view; checkCIF report

## Figures and Tables

**Table 1:** Hydrogen-bond geometry (Å, °) *Cg*1 is the centroid of the C2–C7 benzene ring.

*D*—H⋯*A*	*D*—H	H⋯*A*	*D*⋯*A*	*D*—H⋯*A*
O1—H1⋯O1^i^	0.94 (3)	1.79 (2)	2.7235 (14)	172 (2)
O2—H2⋯N1	0.93 (2)	1.74 (2)	2.5990 (15)	151.7 (19)
C7—H7⋯O1^ii^	0.95	2.57	3.4288 (16)	150
C8—H8⋯O2^iii^	0.95	2.59	3.4492 (16)	151
C1—H1*B*⋯*Cg*1^iv^	0.99	2.56	3.5050 (15)	160

Symmetry codes: (i) 
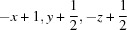
; (ii) 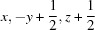; (iii) 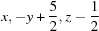; (iv) 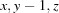.

**Fig. 1.**
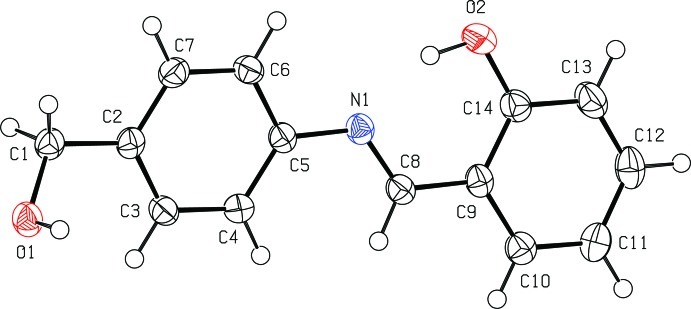
A view of the title compound with the atom numbering scheme. Displacement ellipsoids for non-H atoms are drawn at the 50% probability level.

**Fig. 2.**
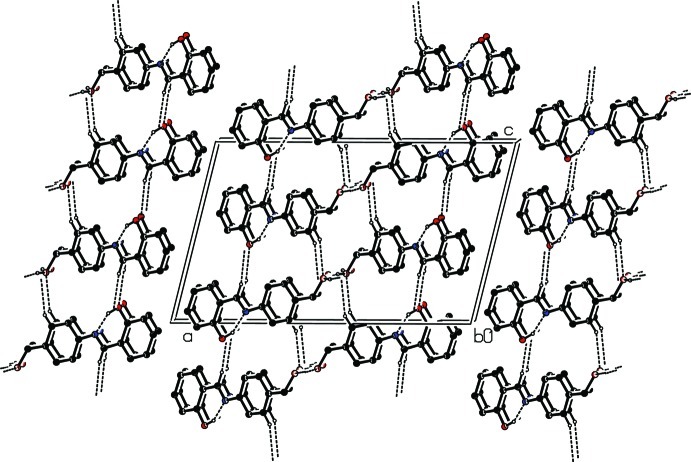
The crystal packing and hydrogen bonding of (I), viewed along the b axis. H atoms not involved in hydrogen bonds have been omitted for clarity.

## supplementary crystallographic information

### Comment

Schiff base compounds are important class of materials due to their flexibility, structural similarities with natural biological substances and also due to presence of imine (–N=CH–) which relates to the mechanism of transformation and racemisation reactions in biological system (Rajavel *et al.*, 2008). Schiff bases-bimolecular condensation products of amnio alcohols with aldehydes represent valuable intermediates in organic synthesis with various applications (Uğraş *et al.*, 2006). Schiff bases resulted from aromatic aldehydes *ortho*-substituted with a hydroxyl group initially aroused interest due to the several donor atoms in their structures which give them an advantage to form a water soluble transition metal complexes (Wadher *et al.*, 2009). This advantage raises potential applications in water treatment (Elena *et al.*, 2000). They could also act as valuable ligands whose biological activity has been shown to increase on complexation (Mohamed *et al.*, 2006).

As seen in Fig. 1, the title compound shows the enol-imine tautomeric form, which has an intramolecular O— H···N hydrogen bond forming an S(6) ring motif (Bernstein *et al.*, 1995). The C14—O2 single bond [1.3525 (17) Å] and the C8═N1 double bond [1.2829 (17) Å] verify the enol-imine form. These distances and the values of the other geometric parameters are in the normal range and are comparable with those of other similar compounds reported previously (Koşar, *et al.*, 2010; Deveci *et al.*, 2008; Ünver *et al.*, 2002; Karadayı *et al.*, 2003). The N1—C8—C9—C14 torsion angle is 1.8 (2)°. Therefore, the N1/C8/C9/C14/O2/H2 S(6) ring is essentially coplanar with the C9–C14 benzene ring to which it is bonded.

In the crystal, molecules are linked by O—H···O and weak C—H···O hydrogen bonds, forming a two dimensional array that stacks along the *a* axis Fig. 2 and Table 1. The crystal packing is further stabilized by C—H···π interactions, Table 1.

### Experimental

The title compound was synthesized as a secondary product from a three component reaction of cyclohexane-1,3-dione (1 mmol), (4-aminophenyl)methanol (1 mmol), and salicylaldehde (1 mmol). The reaction mixture was refluxed in ethanol at 351 K for four hours then left at room temperature for two days. The resulting solid product was filtered of, dried and recrystallized from ethanol. (43% yield, *M*.pt: 403 K). Crystals suitable for X-ray diffraction were grown in a diluted ethanol solution at room temperature by the slow evaporation method.

### Refinement

The H atoms of the hydroxyl groups were located from a difference Fourier map and refined freely [O1—H1 = 0.94 (3) Å and O2—H2 = 0.93 (2) Å]. The hydrogen atoms at C were located geometrically and refined using a riding model with C—H = 0.95 Å for aromatic and 0.99 Å for methylene, and with *U*_iso_ = 1.2*U*_eq_(C). Sixteen poorly fitted reflections (-3 2 10), (-11 0 10), (1 0 0), (-5 1 13), (-7 0 14), (-9 0 14), (-13 1 12), (-12 0 12), (-3 1 14), (-14 1 12), (-8 0 14), (-16 1 12), (-10 5 2), (-4 0 14), (17 1 6), and (12 0 10) were omitted from the refinement.

### Crystal data

**Table:**

C_14_H_13_NO_2_	*F*(000) = 480
*M**_r_* = 227.25	*D*_x_ = 1.351 Mg m^−^^3^
Monoclinic, *P*2_1_/*c*	Cu *K*α radiation, λ = 1.54178 Å
Hall symbol: -P 2ybc	Cell parameters from 11657 reflections
*a* = 19.8172 (14) Å	θ = 2–66°
*b* = 4.7217 (1) Å	µ = 0.73 mm^−^^1^
*c* = 12.3106 (2) Å	*T* = 150 K
β = 104.005 (7)°	Plate, yellow
*V* = 1117.67 (9) Å^3^	0.25 × 0.20 × 0.08 mm
*Z* = 4	

### Data collection

**Table:**

Rigaku RAPID II diffractometer	1641 reflections with *I* > 2σ(*I*)
Confocal optics monochromator	*R*_int_ = 0.030
ω scans	θ_max_ = 66.6°, θ_min_ = 4.6°
Absorption correction: multi-scan (*CrystalClear*; Rigaku/MSC, 2001)	*h* = −23→23
*T*_min_ = 0.838, *T*_max_ = 0.944	*k* = −5→5
10711 measured reflections	*l* = −11→14
1958 independent reflections	

### Refinement

**Table:**

Refinement on *F*^2^	Secondary atom site location: difference Fourier map
Least-squares matrix: full	Hydrogen site location: inferred from neighbouring sites
*R*[*F*^2^ > 2σ(*F*^2^)] = 0.037	H atoms treated by a mixture of independent and constrained refinement
*wR*(*F*^2^) = 0.107	*w* = 1/[σ^2^(*F*_o_^2^) + (0.0585*P*)^2^ + 0.170*P*] where *P* = (*F*_o_^2^ + 2*F*_c_^2^)/3
*S* = 1.14	(Δ/σ)_max_ < 0.001
1958 reflections	Δρ_max_ = 0.23 e Å^−^^3^
163 parameters	Δρ_min_ = −0.18 e Å^−^^3^
0 restraints	Extinction correction: *SHELXL97* (Sheldrick, 2008), FC^*^=KFC[1+0.001XFC^2^Λ^3^/SIN(2Θ)]^-1/4^
Primary atom site location: structure-invariant direct methods	Extinction coefficient: 0.0077 (8)

### Special details

**Table:**

Geometry. Bond distances, angles *etc*. have been calculated using the rounded fractional coordinates. All su's are estimated from the variances of the (full) variance-covariance matrix. The cell e.s.d.'s are taken into account in the estimation of distances, angles and torsion angles
Refinement. Refinement on *F*^2^ for ALL reflections except those flagged by the user for potential systematic errors. Weighted *R*-factors *wR* and all goodnesses of fit *S* are based on *F*^2^, conventional *R*-factors *R* are based on *F*, with *F* set to zero for negative *F*^2^. The observed criterion of *F*^2^ > σ(*F*^2^) is used only for calculating -*R*-factor-obs *etc*. and is not relevant to the choice of reflections for refinement. *R*-factors based on *F*^2^ are statistically about twice as large as those based on *F*, and *R*-factors based on ALL data will be even larger.

### Fractional atomic coordinates and isotropic or equivalent isotropic displacement parameters (Å^2^)

**Table:**

	*x*	*y*	*z*	*U*_iso_*/*U*_eq_	
O1	0.46733 (5)	0.1618 (2)	0.25819 (8)	0.0330 (3)	
O2	0.19567 (6)	1.2380 (2)	0.56950 (8)	0.0408 (4)	
N1	0.24780 (5)	0.9636 (2)	0.42590 (9)	0.0258 (3)	
C1	0.44786 (7)	0.1429 (3)	0.36158 (11)	0.0285 (4)	
C2	0.39457 (6)	0.3581 (3)	0.37420 (11)	0.0252 (4)	
C3	0.34633 (7)	0.4685 (3)	0.28219 (11)	0.0278 (4)	
C4	0.29742 (7)	0.6676 (3)	0.29556 (11)	0.0279 (4)	
C5	0.29508 (6)	0.7592 (3)	0.40265 (11)	0.0245 (4)	
C6	0.34194 (7)	0.6431 (3)	0.49448 (11)	0.0279 (4)	
C7	0.39100 (7)	0.4468 (3)	0.48021 (11)	0.0287 (4)	
C8	0.20820 (6)	1.1083 (3)	0.34759 (11)	0.0257 (4)	
C9	0.15921 (6)	1.3145 (3)	0.37087 (11)	0.0259 (4)	
C10	0.11604 (6)	1.4634 (3)	0.28242 (11)	0.0296 (4)	
C11	0.06849 (7)	1.6600 (3)	0.30125 (13)	0.0340 (4)	
C12	0.06377 (7)	1.7098 (3)	0.41032 (13)	0.0371 (5)	
C13	0.10585 (7)	1.5687 (3)	0.49924 (12)	0.0375 (5)	
C14	0.15422 (7)	1.3713 (3)	0.48079 (11)	0.0299 (4)	
H1	0.4926 (14)	0.330 (5)	0.259 (2)	0.099 (8)*	
H1A	0.48990	0.16730	0.42350	0.0340*	
H1B	0.42910	−0.04890	0.36840	0.0340*	
H2	0.2230 (10)	1.115 (5)	0.5393 (17)	0.071 (6)*	
H3	0.34690	0.40630	0.20900	0.0330*	
H4	0.26540	0.74190	0.23170	0.0330*	
H6	0.34030	0.69910	0.56790	0.0340*	
H7	0.42280	0.37140	0.54410	0.0340*	
H8	0.21100	1.07970	0.27240	0.0310*	
H10	0.11950	1.42860	0.20800	0.0360*	
H11	0.03950	1.75950	0.24050	0.0410*	
H12	0.03090	1.84360	0.42380	0.0440*	
H13	0.10190	1.60590	0.57330	0.0450*	

### Atomic displacement parameters (Å^2^)

**Table:**

	*U*^11^	*U*^22^	*U*^33^	*U*^12^	*U*^13^	*U*^23^
O1	0.0382 (6)	0.0280 (5)	0.0374 (6)	0.0013 (4)	0.0182 (4)	0.0000 (4)
O2	0.0522 (7)	0.0451 (7)	0.0276 (6)	0.0127 (5)	0.0147 (5)	0.0002 (5)
N1	0.0276 (6)	0.0231 (6)	0.0285 (6)	−0.0012 (4)	0.0104 (5)	−0.0012 (4)
C1	0.0317 (7)	0.0239 (7)	0.0317 (7)	−0.0005 (5)	0.0113 (6)	0.0021 (5)
C2	0.0270 (7)	0.0198 (6)	0.0305 (7)	−0.0041 (5)	0.0102 (6)	0.0012 (5)
C3	0.0331 (7)	0.0266 (7)	0.0255 (7)	−0.0008 (5)	0.0105 (6)	−0.0023 (5)
C4	0.0301 (7)	0.0283 (7)	0.0248 (7)	0.0021 (5)	0.0059 (6)	0.0010 (5)
C5	0.0272 (7)	0.0209 (6)	0.0275 (7)	−0.0034 (5)	0.0110 (6)	−0.0013 (5)
C6	0.0354 (7)	0.0264 (7)	0.0236 (7)	−0.0019 (5)	0.0102 (6)	−0.0013 (5)
C7	0.0319 (7)	0.0256 (7)	0.0277 (7)	−0.0006 (6)	0.0054 (6)	0.0025 (5)
C8	0.0289 (7)	0.0235 (7)	0.0263 (7)	−0.0042 (5)	0.0101 (6)	−0.0025 (5)
C9	0.0265 (7)	0.0212 (6)	0.0323 (7)	−0.0041 (5)	0.0116 (6)	−0.0025 (5)
C10	0.0303 (7)	0.0279 (7)	0.0315 (7)	−0.0024 (5)	0.0090 (6)	−0.0015 (6)
C11	0.0291 (7)	0.0275 (7)	0.0451 (9)	0.0001 (6)	0.0085 (6)	0.0008 (6)
C12	0.0326 (8)	0.0302 (8)	0.0528 (10)	0.0022 (6)	0.0189 (7)	−0.0049 (7)
C13	0.0445 (8)	0.0356 (8)	0.0383 (8)	0.0005 (7)	0.0213 (7)	−0.0063 (7)
C14	0.0329 (7)	0.0283 (7)	0.0311 (7)	−0.0018 (5)	0.0130 (6)	−0.0010 (6)

### Geometric parameters (Å, º)

**Table:**

O1—C1	1.4193 (17)	C10—C11	1.382 (2)
O2—C14	1.3525 (17)	C11—C12	1.388 (2)
O1—H1	0.94 (3)	C12—C13	1.377 (2)
O2—H2	0.93 (2)	C13—C14	1.395 (2)
N1—C5	1.4216 (17)	C1—H1A	0.9900
N1—C8	1.2829 (17)	C1—H1B	0.9900
C1—C2	1.5004 (19)	C3—H3	0.9500
C2—C7	1.3886 (19)	C4—H4	0.9500
C2—C3	1.3945 (19)	C6—H6	0.9500
C3—C4	1.388 (2)	C7—H7	0.9500
C4—C5	1.3989 (19)	C8—H8	0.9500
C5—C6	1.3902 (19)	C10—H10	0.9500
C6—C7	1.385 (2)	C11—H11	0.9500
C8—C9	1.4516 (19)	C12—H12	0.9500
C9—C14	1.4063 (19)	C13—H13	0.9500
C9—C10	1.4003 (19)		
			
C1—O1—H1	107.7 (15)	O2—C14—C13	119.12 (12)
C14—O2—H2	105.4 (13)	O1—C1—H1A	109.00
C5—N1—C8	121.56 (11)	O1—C1—H1B	109.00
O1—C1—C2	113.65 (11)	C2—C1—H1A	109.00
C1—C2—C7	119.92 (12)	C2—C1—H1B	109.00
C3—C2—C7	118.02 (12)	H1A—C1—H1B	108.00
C1—C2—C3	122.03 (12)	C2—C3—H3	119.00
C2—C3—C4	121.19 (12)	C4—C3—H3	119.00
C3—C4—C5	120.29 (12)	C3—C4—H4	120.00
N1—C5—C4	124.99 (12)	C5—C4—H4	120.00
C4—C5—C6	118.46 (12)	C5—C6—H6	120.00
N1—C5—C6	116.56 (12)	C7—C6—H6	120.00
C5—C6—C7	120.80 (12)	C2—C7—H7	119.00
C2—C7—C6	121.20 (12)	C6—C7—H7	119.00
N1—C8—C9	121.70 (12)	N1—C8—H8	119.00
C10—C9—C14	118.72 (12)	C9—C8—H8	119.00
C8—C9—C10	119.68 (12)	C9—C10—H10	119.00
C8—C9—C14	121.60 (12)	C11—C10—H10	119.00
C9—C10—C11	121.33 (13)	C10—C11—H11	120.00
C10—C11—C12	118.97 (13)	C12—C11—H11	121.00
C11—C12—C13	121.15 (13)	C11—C12—H12	119.00
C12—C13—C14	120.12 (13)	C13—C12—H12	119.00
C9—C14—C13	119.70 (13)	C12—C13—H13	120.00
O2—C14—C9	121.19 (12)	C14—C13—H13	120.00
			
C5—N1—C8—C9	179.17 (12)	C5—C6—C7—C2	−0.6 (2)
C8—N1—C5—C4	−8.8 (2)	N1—C8—C9—C10	−178.71 (12)
C8—N1—C5—C6	171.49 (12)	N1—C8—C9—C14	1.8 (2)
O1—C1—C2—C7	−153.49 (12)	C8—C9—C10—C11	179.61 (13)
O1—C1—C2—C3	28.42 (18)	C14—C9—C10—C11	−0.9 (2)
C3—C2—C7—C6	−1.2 (2)	C8—C9—C14—O2	0.9 (2)
C1—C2—C3—C4	−179.98 (14)	C8—C9—C14—C13	−179.25 (13)
C1—C2—C7—C6	−179.38 (13)	C10—C9—C14—O2	−178.60 (12)
C7—C2—C3—C4	1.9 (2)	C10—C9—C14—C13	1.3 (2)
C2—C3—C4—C5	−0.8 (2)	C9—C10—C11—C12	0.0 (2)
C3—C4—C5—C6	−1.0 (2)	C10—C11—C12—C13	0.5 (2)
C3—C4—C5—N1	179.32 (13)	C11—C12—C13—C14	−0.1 (2)
C4—C5—C6—C7	1.7 (2)	C12—C13—C14—O2	179.11 (13)
N1—C5—C6—C7	−178.61 (12)	C12—C13—C14—C9	−0.8 (2)

### Hydrogen-bond geometry (Å, º)

Cg1 is the centroid of the C2–C7 benzene ring.

**Table:**

*D*—H···*A*	*D*—H	H···*A*	*D*···*A*	*D*—H···*A*
O1—H1···O1^i^	0.94 (3)	1.79 (2)	2.7235 (14)	172 (2)
O2—H2···N1	0.93 (2)	1.74 (2)	2.5990 (15)	151.7 (19)
C7—H7···O1^ii^	0.95	2.57	3.4288 (16)	150
C8—H8···O2^iii^	0.95	2.59	3.4492 (16)	151
C1—H1*B*···*Cg*1^iv^	0.99	2.56	3.5050 (15)	160

Symmetry codes: (i) −*x*+1, *y*+1/2, −*z*+1/2; (ii) *x*, −*y*+1/2, *z*+1/2; (iii) *x*, −*y*+5/2, *z*−1/2; (iv) *x*, *y*−1, *z*.
